# Long-Term Impact of Interdisciplinary Collaboration on Oral Health in Children with Nephrotic Syndrome: A 12-Year Retrospective Study

**DOI:** 10.3390/jcm14082696

**Published:** 2025-04-15

**Authors:** Paula Piekoszewska-Ziętek, Małgorzata Pańczyk-Tomaszewska, Dorota Olczak-Kowalczyk

**Affiliations:** 1 Department of Paediatric Dentistry, Medical University of Warsaw, 02-091 Warsaw, Poland; 2 Department of Paediatrics and Nephrology, Medical University of Warsaw, 02-091 Warsaw, Poland; mpanczyk1@wum.edu.pl

**Keywords:** nephrotic syndrome, oral health, pediatric dentistry, caries, gingivitis

## Abstract

**Objectives**: Children with nephrotic syndrome (NS) are prone to oral health issues due to immunosuppression and systemic inflammation, which may exacerbate their renal condition. The objective of this study was to evaluate the impact of a 12-year interdisciplinary collaboration between pediatric dentists and nephrologists on oral health in children with NS. **Methods**: A retrospective analysis of 80 NS patients—40 assessed in 2012 and 40 in 2024—was conducted using caries indices (dmft/DMFT), Plaque Index, and Gingival Index. Statistical tests assessed differences between groups (*p* < 0.05). **Results**: The prevalence of active caries significantly decreased (50% vs. 78%; *p* = 0.011), with fewer decayed permanent teeth (0.96 ± 1.56 vs. 2.66 ± 2.51; *p* = 0.003) and improved oral hygiene (good hygiene in 52.5% vs. 30%; *p* = 0.041) in the 2024 group. Gingivitis was less severe compared to 2012. **Conclusions**: Long-term interdisciplinary care significantly improved oral health in children with NS. These improvements may contribute to reduced systemic inflammation and better overall disease control. Integrating dental care into NS management is recommended to support long-term outcomes.

## 1. Introduction

Nephrotic syndrome (NS) is a clinical condition characterized by significant proteinuria that exceeds the individual’s ability to compensate (greater than 50 mg/kg/day), resulting from increased glomerular permeability to plasma proteins. A hallmark feature of NS is its tendency to recur, sometimes as frequently as every few months [1,2]. In children, the majority of cases are classified as idiopathic nephrotic syndrome, which is typically associated with minimal change disease and managed with glucocorticoid therapy [3,4]. Although the etiology of NS remains unclear, immune and inflammatory factors are thought to play an important role. These mechanisms can lead to damage to the glomerular barrier, resulting in pathological protein seepage into the urine [5].

Patients with nephrotic syndrome require special attention to their overall health, as the inflammatory conditions are associated with a number of complications that can impair quality of life and accelerate the progression of kidney damage. Proper management of general health is major not only to better control symptoms, but also to reduce the risk of complications and improve long-term prognosis [6]. Affected with NS, especially those taking immunosuppressive medication (e.g., glucocorticosteroids), are more prone to infections, including those developing in the oral cavity [7]. Inflammatory conditions in the oral cavity, especially those caused by untreated chronic diseases such as caries, periodontal disease, or abscesses, can significantly affect the course of NS. The oral cavity is considered a potential source of inflammatory foci that can lead to a systemic response [8]. These mechanisms include activation of the immune system and secretion of pro-inflammatory cytokines, such as interleukin-6 (IL-6) or -18 (IL-18) and tumor necrosis factor-alpha (TNF-α), which have been documented to exacerbate kidney disease [9,10]. Bacterial endotoxins, such as lipopolysaccharides released from periodontal pathogens, can enter the bloodstream through ulcerated or inflamed oral tissues, leading to transient or chronic low-grade bacteremia [11,12]. This process activates innate immune receptors, including Toll-like receptor 4 (TLR-4), on circulating immune cells and renal podocytes, triggering the release of pro-inflammatory cytokines [9,10]. These mediators have been implicated in increased glomerular permeability and podocyte injury, contributing to proteinuria—a hallmark of nephrotic syndrome [10,13]. In children with NS, where immune regulation is already disturbed due to the disease and immunosuppressive treatment, oral-derived inflammatory signals may act as additional triggers, potentially worsening disease activity or provoking relapses. Thus, maintaining oral health may represent a modifiable factor in reducing systemic inflammation and supporting the clinical stability of nephrotic syndrome. Repeated episodes of bacteremia can activate the immune system and increase the inflammatory process in the kidneys, contributing to the aggravation of NS. There is also evidence that bacterial toxins, such as lipopolysaccharides, can directly damage glomerular epithelial cells [11,12].

Oral inflammations are an important risk factor for patients with nephrotic syndrome. Regular monitoring of oral health is crucial in children with NS. Proper oral hygiene, regular dental examinations, and treatment of caries and oral infections can help reduce the risk of disease exacerbation and reduce proteinuria during the relapse [12,14]. It is recommended that children with TMD be under constant dental care, including prevention of dental caries and periodontal disease’s early detection of inflammatory foci, allowing them to avoid complications and improve their quality of life [15,16].

In the Academic Year 2011–2012, a collaboration was established between the Department of Pediatric Dentistry and the Department of Pediatrics and Nephrology, Medical University of Warsaw, to monitor oral health and motivate dental treatment and behavioral change. Therefore, the purpose of this study was to compare the dental and gingival health of children treated for nephrotic syndrome at the beginning and after 12 years of cooperation between the Departments.

## 2. Materials and Methods

A positive opinion was obtained from the Bioethics Committee of the Medical University of Warsaw for the study conducted in 2012 (KB/187/2011, approval date: 13 December 2011) and for the study conducted in 2024 (KB/26/2020, approval date: 3 February 2020). This is a retrospective, comparative, cross-sectional study. A review of medical records was carried out for patients treated for nephrotic syndrome at the Department of Pediatrics and Nephrology, Medical University of Warsaw, who, as part of an interdepartmental collaboration, were referred for examination and potential treatment at the Department of Pediatric Dentistry, Medical University of Warsaw. The collaboration’s purpose was to provide comprehensive oral health monitoring for children with nephrotic syndrome, promote preventive behaviors, and facilitate access to dental care. Patients were systematically referred for oral examination, caries risk assessment, and individualized preventive or restorative treatment. Educational materials were also provided to families as part of the joint effort to reduce oral inflammation and related systemic risks. The control group consisted of patients treated in 2012 (the beginning of the collaboration), while the study group comprised patients treated in 2024 (after 12 years of collaboration). The primary outcome was the comparison of dental caries prevalence, severity (dmft/DMFT), and oral hygiene status (Plaque Index) between the two cohorts. Secondary outcomes included the presence of active carious lesions, white spot lesions, and gingival health status (Gingival Index). Only complete patient data were included in the study, while incomplete data were disqualified. Ultimately, data from 80 patients were included, with 40 patients in both the study and control groups.

The following data were extracted from patient’s medical records:

- Age at the time of treatment, sex;
- Type of dentition (primary/mixed/permanent);
- Number of primary and permanent teeth with white spot lesions (ICDAS-II code 1–2 [17]);
- Presence of active dental caries;
- Values of the dmft/DMFT Indices (decayed-missing-filled teeth in primary dentition/decayed-missing-filled teeth in permanent dentition) and their individual components;
- Plaque Index value [18] and its interpretation;
- Gingival Index value [18] and its interpretation.

The extracted data were organized, compiled into tables, and subjected to statistical analysis. The following statistical tests were applied: for qualitative data, χ^2^ tests; for quantitative data, the Student’s *t*-tests for independent samples or Mann–Whitney U tests, as well as the Student’s *t*-tests for independent samples. The significance level was set at *p* < 0.05. The effect size was determined as follows: for qualitative data, Cramér’s V coefficient; for quantitative data, Cohen’s d coefficient or the r coefficient.

## 3. Results

Records from 80 patients were analyzed, including 52 boys and 28 girls. The mean age of the patients was 10.0 ± 4.53 years. No significant differences were found between the groups regarding sex (*p* = 0.879) or age (*p* = 0.481). The characteristics of the groups are provided in Table 1.

In the study group, 34 cases of dental caries were recorded (85%), compared to 36 cases in the control group (90%) (*p* = 0.49). However, the number of patients with active carious lesions significantly decreased over the observed period, with 20 cases (50%) in the study group and 31 cases (78%) in the control group (*p* = 0.011).

For primary teeth, the study group exhibited comparable or lower values of the dmft Index and its individual components (d—decayed milk teeth; m—missing milk teeth; f—filled milk teeth). Slight differences were also observed in the mean number of teeth with white spot lesions. However, these differences were not statistically significant. The data are summarized in Table 2.

In permanent dentition, the study group exhibited lower values of the DMFT Index and its individual components (D—decayed permanent teeth; M—missing permanent teeth; F—filled permanent teeth). Significant results were observed in the mean number of permanent teeth with active carious lesions (2.66 ± 2.51 vs. 0.96 ± 1.56; *p* = 0.003). The study group also had a lower mean number of permanent teeth with white spot lesions. Detailed data are presented in Table 3.

After 12 years of collaboration, a trend toward improved oral hygiene and, consequently, better gingival health was observed among patients. A significantly higher percentage of patients in the study group had good oral hygiene (PL.I. < 1) compared to the control group (52.5% vs. 30%; *p* = 0.041). A decrease in the values of the Plaque Index and Gingival Index was also noted; however, these differences were not statistically significant. Moderate gingivitis was statistically more frequent in the control group (*p* = 0.033). These data are summarized in Table 4.

## 4. Discussion

In this retrospective study, we observed that after 12 years of interdisciplinary collaboration between pediatric dentistry and pediatric nephrology departments, children with nephrotic syndrome showed notable improvements in oral health indicators. Specifically, the study group exhibited a significantly lower number of active carious lesions, fewer decayed permanent teeth, and better oral hygiene status compared to the control group from 2012. Although the overall prevalence of dental caries remained high, the reduction in active disease and gingival inflammation suggests a positive trend in oral health outcomes. These findings highlight the potential benefits of integrated care models for medically compromised pediatric populations. Studies by Luong et al. [16] and Kaczmarek et al. [14] reported significantly worse oral health in children with nephrotic syndrome compared to healthy peers. These studies emphasized the elevated risk of caries, gingival inflammation, and enamel defects in NS patients, underscoring the need for targeted dental care. Unlike their cross-sectional designs, our study evaluated oral health status over a 12-year period and demonstrated that proactive, interdisciplinary collaboration can lead to tangible improvements.

Idiopathic nephrotic syndrome is the most common glomerular disease in children, yet it remains relatively rare, with an incidence ranging from 1.4 to 6.1 per 100,000 children, depending on ethnicity [19]. The majority of affected children respond to standard oral corticosteroid therapy and are classified as having steroid-sensitive nephrotic syndrome. While most patients experience relapses throughout childhood, disease progression varies—35–40% have only a single episode or one to two relapses, whereas 55–60% experience more frequent relapses [6]. Steroid resistance occurs in 5–15% of cases, making management more challenging. Steroid-sensitive patients undergo cycles of remission and relapse, necessitating repeated courses of corticosteroids. However, prolonged steroid use can lead to cumulative adverse effects [6,20].

Complications of idiopathic nephrotic syndrome include acute kidney injury, thromboembolic events (1.5–3.8% in children), and life-threatening hypovolaemia, particularly during relapses when albumin levels drop below 20–25 g/L. Severe edema, shock, and pulmonary edema may occur, requiring careful fluid management, albumin infusion, and cautious diuretic use. Additionally, infections pose a major risk due to urinary loss of immunoglobulins and immunosuppressive therapy, increasing susceptibility to sepsis, pneumonia, and peritonitis, which necessitate early detection and treatment [21,22].

Children with nephrotic syndrome are susceptible to various oral health complications due to both the disease and its treatment. Studies have shown that children with NS have a higher risk of gingivitis, gingival overgrowth, dental caries, and developmental defects of enamel (DDE) compared to healthy controls. The increased prevalence of gingival inflammation and overgrowth is often associated with immunosuppressive therapies, such as cyclosporine and nifedipine, which are commonly used in NS management [16]. Additionally, disturbances in calcium and phosphate metabolism inherent to NS can lead to DDE, further compromising dental integrity [23]. Untreated dental conditions, such as caries, pulp diseases, or periapical inflammation, can exacerbate the course of nephrotic syndrome and potentially trigger relapses. Infections are a well-recognized precipitant of nephrotic syndrome relapses, as they can stimulate immune responses that increase glomerular permeability, leading to proteinuria [24]. Oral infections, including dental caries and periodontal disease, may serve as persistent sources of inflammation and infection, thereby increasing the risk of relapse in nephrotic syndrome patients [25]. Therefore, maintaining optimal oral health through regular dental care is crucial in managing nephrotic syndrome and reducing the likelihood of disease exacerbation.

Beyond the role of pro-inflammatory cytokines, recent studies have identified more specific pathways linking systemic inflammation to glomerular injury in nephrotic syndrome. For instance, the activation of TLR4 by circulating lipopolysaccharides may lead to upregulation of nuclear factor kappa B (NF-κB) in podocytes, promoting cytoskeletal disorganization and loss of slit diaphragm integrity, which are key features in proteinuria development [13,26]. Additionally, elevated levels of IL-18, a cytokine often associated with oral and periodontal inflammation, have been shown to correlate with disease activity in NS and serve as a potential biomarker of tubular injury [9]. Systemic low-grade inflammation can also affect endothelial glycocalyx function, impairing glomerular barrier selectivity and further promoting protein leakage. While our study did not assess molecular markers directly, the observed improvement in clinical oral parameters may reflect a reduction in these subclinical inflammatory processes. Incorporating biomarker analysis (e.g., IL-6, IL-18, NGAL, and KIM-1) in future longitudinal studies could provide more direct evidence for this mechanistic connection and help quantify the systemic impact of oral health interventions in nephrotic patients.

To the best of our knowledge, no previous study has been published assessing the impact of interdisciplinary collaboration between dentists and nephrologists in the management of patients with nephrotic syndrome. Previous studies rather compared diseased patients with generally healthy controls. A study by Kaczmarek et al. [14] revealed that NS patients, compared to the controls, were exposed to some lower caries experience (83.0% vs. 95.7%), a significantly lower number of filled primary and/or permanent teeth (1.1 ± 1.6 vs. 3.5 ± 3.0, *p* < 0.001), and higher incidence of enamel hypoplasia (31.9% vs. 4.3%, *p* < 0.001). Patients with NS had higher OHI-S and GI scores. Luong et al. [16] reported that NS patients showed significantly higher scores of OHI-S, GI, and dmft, and higher proportions of dental caries and DDE than those of the controls (*p* < 0.001). This highlights the necessity of educating these patients, raising awareness about the consequences of oral inflammation on the progression of systemic disease, and ensuring the implementation of effective, individualized treatment strategies. On the contrary to those findings, a recent paper by Beyer et al. [27] found no significant differences in oral health status across renal conditions, suggesting that access to consistent dental care—rather than diagnosis alone—may be a key determinant in oral outcomes. Our results support this perspective and provide evidence that structured collaboration may mitigate the typical oral health disadvantages observed in children with chronic kidney disease.

Our study demonstrated that 12 years of collaboration between pediatric nephrologists and pediatric dentists resulted in significant improvements in oral health, which may positively impact the overall health of children with nephrotic syndrome. This confirms that long-term interdisciplinary collaboration between nephrologists and dentists improves oral hygiene, reduces caries prevalence, and lowers gingival inflammation in children with nephrotic syndrome. Since oral infections can exacerbate NS and contribute to relapses, regular dental care should become a standard component of treatment and prevention strategies for pediatric NS patients.

Effective dental care for patients with nephrotic syndrome requires close collaboration between dentists and pediatric nephrologists, as well as a thorough understanding of the interplay between systemic and oral health issues. Due to the chronic nature of the disease, maintaining good oral health is essential. For most patients with idiopathic nephrotic syndrome, the condition does not hinder routine dental care, and treatments can generally be performed regularly during remission periods. However, surgical procedures should be undertaken with appropriate antibiotic prophylaxis and in consultation with a nephrologist.

Given the nature of the research, it has some limitations. The study relies on a retrospective review of medical records, which may introduce selection bias as only complete records were included. Incomplete data were disqualified, potentially excluding patients with more severe conditions or less consistent follow-up. The findings may not be applicable to other populations, healthcare systems, or geographic locations. Differences in healthcare access, socioeconomic status, and diet could influence the results of a multicenter study. While the study provides valuable insights into the oral health status of children with nephrotic syndrome over a 12-year period, its retrospective design, quite a small sample size, and single-center nature limit its ability to establish a direct causal relationship between collaboration and improved oral health. Furthermore, potential confounding factors related to the 12-year time gap between study groups were not evaluated. Advancements in nephrotic syndrome treatment protocols, broader improvements in health education, and increased public awareness regarding oral hygiene may have independently contributed to the observed improvements. Additionally, systemic changes in access to dental care or evolving parental attitudes toward preventive health may have influenced patient behavior, regardless of our interdepartmental collaboration. These variables could not be controlled in the retrospective study design and were not systematically recorded in patient files. Future studies should focus on prospective, multicenter studies with larger sample sizes and control for confounding factors such as medication use, diet, and socioeconomic status and consider incorporating behavioral and systemic factors alongside clinical data to better isolate the effects of collaborative care models on oral-systemic outcomes.

## 5. Conclusions

This 12-year retrospective study suggests that long-term interdisciplinary collaboration between pediatric nephrology and pediatric dentistry can support better oral health outcomes in children with nephrotic syndrome. While caries prevalence remains high, the observed improvements in oral hygiene and reduction in active lesions highlight the value of integrated preventive care. These findings reinforce the need to incorporate dental monitoring into chronic disease management strategies and encourage further prospective studies to explore the systemic impact of such models.

## Figures and Tables

**Table 1:** Characteristics of included patients.

Variable	Study Group (2024, *n* = 40)	Control Group (2012, *n* = 40)	Statistical Test	*p*-Value
Mean age (years ± SD)	10.7 ± 4.44	9.99 ± 4.53	t(78) = 0.71	0.481
Sex—Male (*n*/%)	28 (70%)	24 (60%)	χ^2^(1) = 0.02	0.879
Sex—Female (*n*/%)	12 (30%)	16 (40%)		

**Table 2:** Differences in dmft Index and white spot lesion score (ICDAS-II 1–2) in primary teeth.

	Study Group N = 40	Control Group N = 40	Statistical Significance
Mean number of primary teeth with ICDAS-II 1–2 score	1.41 ± 1.40	1.17 ± 1.27	*t*(44) = −0.61; *p* = 0.542; *d* = 0.18
Mean d	1.45 ± 2.20	2.00 ± 2.89	*U* = 237.5; *Z* = −0.62; *p* = 0.535; *r* = 0.09
Mean m	0.73 ± 1.72	0.71 ± 2.87	*U* = 237; *Z* = −0.90; *p* = 0.369; *r* = 0.13
Mean f	1.64 ± 2.15	2.63 ± 2.37	*t*(44) = 1.48; *p* = 0.147; *d* = 0.44
Mean dmft	3.82 ± 3.66	5.33 ± 4.34	*t*(44) = 1.27; *p* = 0.209; *d* = 0.38

**Table 3:** Differences in DMFT Index and white spot lesion score (ICDAS-II 1–2) in permanent teeth.

	Study Group N = 40	Control Group N = 40	Statistical Significance
Mean number of permanent teeth with ICDAS-II 1–2 score	1.23 ± 1.66	2.64 ± 5.44	*U* = 441.5; *Z* = −0.71; *p* = 0.476; *r* = 0.09
Mean D	0.96 ± 1.56	2.66 ± 2.51	*t*(44) = 3.14; *p* = 0.003 *; *d* = 0.82
Mean M	0 ± 0	0.07 ± 0.26	*U* = 472.5; *Z* = −1.57; *p* = 0.117; *r* = 0.20
Mean F	2.46 ± 4.05	2.69 ± 4.08	*U* = 491.5; *Z* = −0.23; *p* = 0.882; *r* = 0.03
Mean DMFT	3.43 ± 4.12	5.41 ± 4.88	*U* = 380; *Z* = −1.74; *p* = 0.082; *r* = 0.22

* statistically significant.

**Table 4:** Oral hygiene state and gingivitis in the control and study group.

	Study Group N = 40	Control Group N = 40	Statistical Significance
Mean Plaque Index	1.15 ± 0.87	1.22 ± 0.61	*t*(70.17) = 0.42; *p* = 0.675; *d* = 0.09
Number of patients with good oral hygiene *n*/%	21/52.5	12/30	χ^2^(1) = 4.18; *p* = 0.041 *; *V* = 0.23
Number of patients with average or bad oral hygiene *n*/%	19/47.5	28/70	
Mean Gingival Index	0.78 ± 0.79	1.01 ± 0.75	*t*(78) = 1.37; *p* = 0.175; *d* = 0.31
Number of patients with no gingivitis *n*/%	12/30	5/12.5	χ^2^(1) = 3.66; *p* = 0.056; *V* = 0.21
Number of patients with mild gingivitis *n*/%	14/35	12/30	χ^2^(1) = 0.23; *p* = 0.633; *V* = 0.05
Number of patients with moderate gingivitis n/%	9/22.5	18/45	χ^2^(1) = 4.53; *p* = 0.033 *; *V* = 0.24
Number of patients with severe gingivitis n/%	5/12.5	5/12.5	χ^2^(1) = 0; *p* = 1; *V* = 0

* statistically significant.

## Data Availability

The data that support the findings of this study are not openly available due to reasons of sensitivity and are available from the corresponding author upon reasonable request. Data are located in controlled access data storage at the Medical University of Warsaw.
